# *Plasmodium* LCCL domain-containing modular proteins have their origins in the ancestral alveolate

**DOI:** 10.1098/rsob.230451

**Published:** 2024-06-12

**Authors:** Callum De Hoest-Thompson, Virginia Marugan-Hernandez, Johannes T. Dessens

**Affiliations:** ^1^ The Royal Veterinary College, Hawkshead Lane, North Mymms AL9 7TA, UK; ^2^ Department of Infection Biology, Faculty of Infectious and Tropical Diseases, London School of Hygiene & Tropical Medicine, London WC1E 7HT, UK

**Keywords:** malaria, Apicomplexa, Alveolata, Colpenemida

## Abstract

*Plasmodium* species encode a unique set of six modular proteins named LCCL lectin domain adhesive-like proteins (LAPs) that operate as a complex and that are essential for malaria parasite transmission from mosquito to vertebrate. LAPs possess complex architectures obtained through unique assemblies of conserved domains associated with lipid, protein and carbohydrate interactions, including the name-defining LCCL domain. Here, we assessed the prevalence of *Plasmodium* LAP orthologues across eukaryotic life. Our findings show orthologous conservation in all apicomplexans, with lineage-specific repertoires acquired through differential *lap* gene loss and duplication. Besides Apicomplexa, LAPs are found in their closest relatives: the photosynthetic chromerids, which encode the broadest repertoire including a novel membrane-bound LCCL protein. LAPs are notably absent from other alveolate lineages (dinoflagellates, perkinsids and ciliates), but are encoded by predatory colponemids, a sister group to the alveolates. These results reveal that the LAPs are much older than previously thought and pre-date not only the Apicomplexa but the Alveolata altogether.

## 1. Introduction

**Figure 1.**
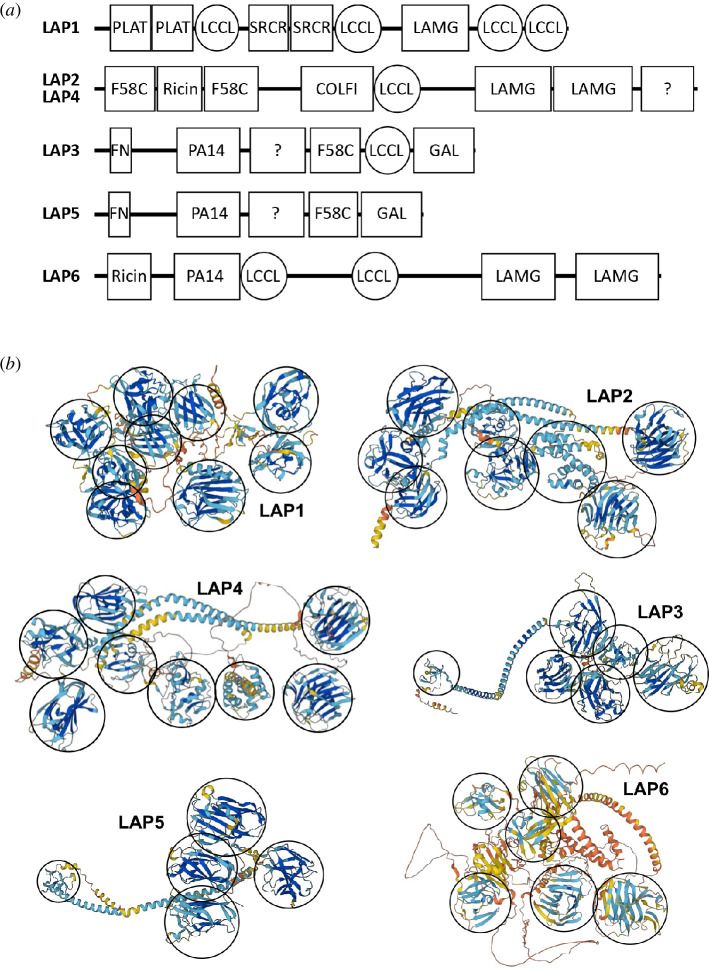
Predicted domain composition and organization of the LAPs. (*a*) Schematic linear depiction of LAP1-LAP6 with various conserved domains indicated: PLAT (polycystin-1, lipoxygenase, alpha-toxin, IPR001024); LCCL (*Limulus* coagulation factor C, Coch-5b2 and Lgl1, IPR004043); SRCR (scavenger receptor cysteine-rich, IPR036772); LAMG (laminin G superfamily, IPR013320, cl22861); F58C (coagulation factor V and VIII carboxy-terminal, IPR000421, related to discoidin); Ricin (ricin B lectin domain, IPR000772); COLFI (fibrillar collagen carboxy-terminal domain, SM00038, PF01410); FN (fibronectin type II domain, IPR000562); PA14 (protective antigen of anthrax toxin, IPR037524); GAL (galactose binding-like domain superfamily, IPR008979); ? (unspecified domains). (*b*) Predicted AlphaFold three-dimensional structure projections of *Plasmodium falciparum* LAP1-LAP6. Protein domains are encircled.

Malaria parasites encode a set of six conserved modular proteins, named LCCL lectin adhesive-like proteins (LAPs), that are essential for parasite transmission from mosquito to vertebrate [1–4]. The LAPs have amino-terminal endoplasmic reticulum signal peptides and possess modular architectures composed of a variety of domains implicated in lipid, protein and carbohydrate binding, including the typifying LCCL domain (IPR004043, named after the metazoan proteins *Limulus* clotting factor C, Coch-5b2 and Lgl1 where the domain was first identified [5]) (figure 1) [1,4,6]. *Plasmodium* LAPs operate as a protein complex and subcellularly co-localize [7–12], demonstrating a functional relatedness accompanying their structural similarities. However, the precise molecular mechanism by which the LAPs operate in *Plasmodium* remains an enigma.

The LAPs were previously reported to be confined to the phylum Apicomplexa and to exhibit conservation across apicomplexan lineages, albeit with possible differences in their repertoires [6,13]. Given the large expansion of genomic and transcriptomic sequence data for both apicomplexan and other organisms since these studies were carried out more than a decade ago, we decided to re-assess the LAPs in terms of their structure, repertoire and distribution across eukaryotic life. The results obtained confirm the presence of apicomplexan lineage-specific LAP repertoires but also reveal complete orthologous conservation of the LAPs in their photosynthetic chromerid relatives. Our surprising discovery of LAPs in colponemids shows the LAPs are much older than previously assumed.

## 2. Results

### 2.1. LAP structure and domain composition

**Figure 2.**
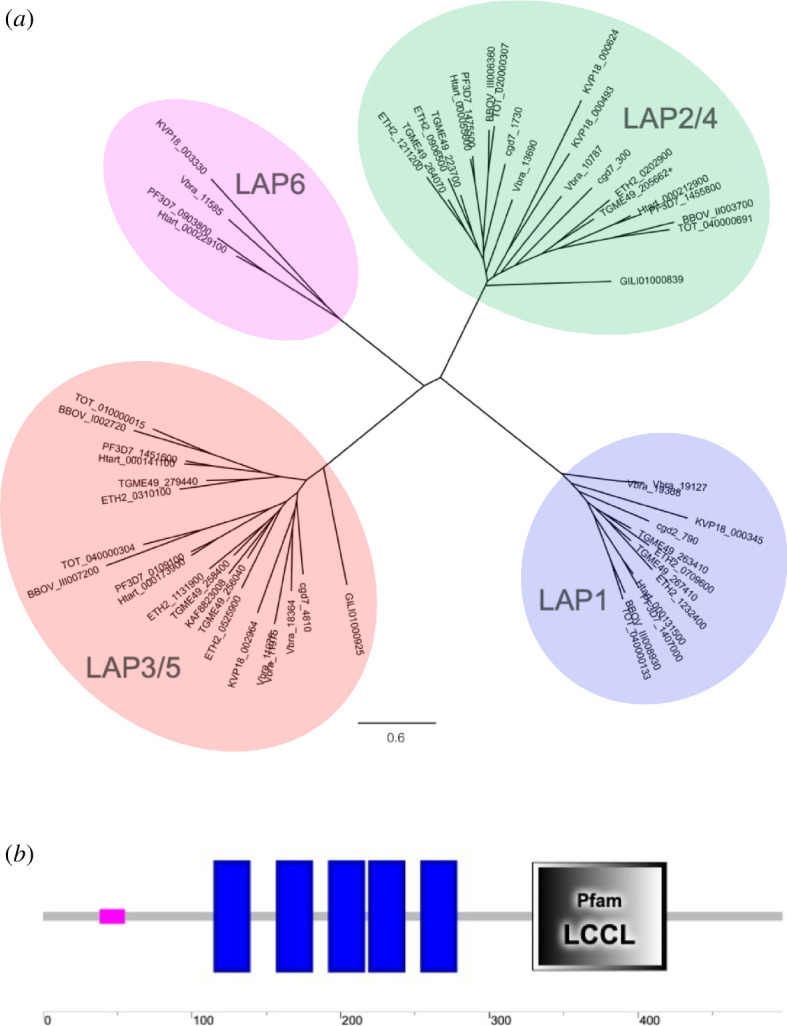
LAPs cluster by type. (*a*) Phylogeny generated from LAP1 type (blue), LAP2/4 type (green), LAP3/5 type (red) and LAP6 type (pink) amino acid sequences identified in apicomplexan, chromerid and colponemid lineages. See electronic supplementary material, table S1 for sequence details. Scale bar: substitutions per site. (*b*) Predicted structure of the membrane-bound LCCL-domain proteins found in chromerids. Blue rectangles represent predicted transmembrane helices (image generated in SMART).

The six LAPs that were first identified in *Plasmodium* have distinct domain compositions and topologies [14]. The exceptions to this are LAP2 and LAP4, which have identical domain composition and order and are, in effect, structural paralogues (figure 1). Similarly, LAP5 has the same domain topology and composition as LAP3 but lacks the LCCL domain and is included in the LAP family by virtue of its paralogous relationship with LAP3 (figure 1). Thus, the six LAPs represent, in fact, only four distinct structural types referred to in this paper as LAP1, LAP2/4, LAP3/5 and LAP6, respectively (figure 2).

During the analysis of LAP orthologues for the presence of conserved domains, it became apparent that recognized LAP domains are not identified in all orthologues. This probably reflects amino acid sequence divergence between orthologues within the structural constraints of maintaining functionality, allowing domains to elude detection in some orthologues. It cannot be ruled out, however, that certain domains in certain orthologues may have lost functionality altogether and are retained solely as a linker to maintain the tertiary and quaternary structures of the LAPs, although this seems less likely given their length. The examination of new LAP orthologues also allowed us to identify further, hitherto undetected, modules in the LAP family (figure 1). For example, in LAP1, we identified a second PLAT domain (polycystin-1, lipoxygenase, alpha-toxin, IPR001024) at the amino terminus (figure 1*a*). We also identified a second F58C domain (coagulation factor V and VIII carboxy-terminal, IPR000421), related to discoidin, at the amino terminus of LAP2/4, as well as two tandem LAMG (laminin G) domains in the carboxy-terminal portion of LAP2/4 (figure 1*a*). The latter domain is structurally related to the concanavalin A-like lectin/glucanase domain superfamily (IPR013320) as well as the LamG superfamily (cl22861) and is found in a variety of animal proteins including pentraxins, laminin G, glycosyl hydrolases and plant lectins. A galactose-binding-like domain (IPR008979, GAL) was newly identified in the carboxy-terminal portion of LAP3/5, and we also discovered a second LAMG domain in the carboxy-terminal portion of LAP6 (figure 1*a*).

Examination of the predicted three-dimensional structures of the LAPs in the AlphaFold Protein Structure Database allowed us to identify more precisely the number and relative positions of discrete protein domains in each of the LAPs (figure 1*b*). Integration of this data with our conserved domain searches revealed the presence of two additional, unspecified protein domains in LAP3/5 and LAP2/4, respectively (figure 1*a*). Protein structure comparison by distance matrix alignment (DALI) of resolved three-dimensional structures of conserved protein domains against the AlphaFold-generated three-dimensional structure predictions of the *Plasmodium falciparum* proteome did not identify additional LCCL domain-containing proteins, but confirmed the presence of LCCL (PDB: 1JBI), F58C (PDB: 3NNG), ricin B (PDB: 4P6A), fibronectin type II (FN) (PDB: 7PRK), protective antigen of anthrax toxin (PA14) (PDB: 4GQ7), LAMG (PDB: 6OAI) and scavenger receptor cysteine-rich (SRCR) (PDB: 5JFB) domains in corresponding LAPs (figure 1*a*). Collectively, these results show that structural conservation closely mirrors sequence conservation in this group of proteins.

Some LAP regions were occasionally recognized as distinct conserved domains by different search algorithms/databases or in different orthologues. For example, the F58C domain overlapped with the GAL domain, as well as with a LamNT domain (laminin N-terminal domain, SM000136). Similarly, the COLFI (fibrillar collagen carboxy-terminal domain, SM00038, PF01410) domain overlapped with a FBG domain (fibrinogen-related domain, SM000186). These observations could reflect a common structural architecture of the domains in question, achieved through common ancestry or convergent evolution. Indeed, the F58C domain forms part of the galactose-binding-like domain superfamily (IPR008979), indicating that it has sugar-binding properties. Furthermore, DALI searches with galectin (PDB: 7XFA) and pea lectin (PDB: 1BQP) identified domains overlapping with the LAMG and GAL domains, indicating these all share a structural scaffold. Overall, the majority of LAP modules appear to possess carbohydrate-binding features.

### 2.2. LAP distribution and repertoires

A range of LAP orthologues were identified in genomes of the main apicomplexan lineages Hematozoa, Coccidia, Cryptosporidia and Gregarinia (electronic supplementary material, table S1). LAP amino acid sequences clustered according to type by phylogenetic methods (figure 2*a*), confirming the correct assignment of type to the various LAP sequences. Distinct LAP repertoires were identified in different apicomplexan lineages (table 1). At least one LAP orthologue of each of types LAP1, LAP2/4 and LAP3/5 was identifiable in all lineages examined, while LAP6 was the least prevalent type and is missing in coccidian and cryptosporidian lineages (table 1). LAP6 is also notably absent in the tick-transmitted Piroplasmida (represented by *Theileria orientalis* and *Babesia bovis*), a notable difference from its closely related insect-transmitted relatives (Haemosporida) (table 1). The two families within the Haemosporida lineage: *Plasmodiidae* (represented by *P. falciparum*) and *Haemoproteidae* (represented by *Haemoproteus tartakovskyi*), have identical LAP repertoires, as is true for the two families in the coccidian lineage: *Eimeriidae* (represented by *Eimeria tenella*) and *Sarcocystidae* (represented by *Toxoplasma gondii*) (table 1). We were unable to evaluate LAP repertoires in the apicomplexan lineages Marosporida and Nephromycida [15] owing to incomplete genome coverage. Nonetheless, we identified a LAP3 in *Cardiosporidium cionae* (Nephromycida) (electronic supplementary material, table S1), as well as putative LAP1, LAP2/4 and LAP3-encoding transcripts in *Nephromyces* (Nephromycida) and *Rhytidocystis* (Marosporida) species (not shown), indicating that LAPs are present in all apicomplexan lineages to date.

**Table 1.** Minimum LAP repertoire in apicomplexan and chromerid lineages.

lineage		LAP type					
		LAP1	LAP2/4	LAP3	LAP5	LAP6	other^a^
Hematozoa	Haemosporida	1	2	1	1	1	0
	Piroplasmida	1	2	1	1	0	0
Coccidia	*Eimeriidae*	2	3	2	1	0	0
	*Sarcocystidae*	2	3	2	1	0	0
Cryptosporidia		1	2	0	1	0	0
Gregarinia		1	2	1	0	1	0
Chromerida		2	2	1	2	1	2

^a^ LCCL domain-containing protein with five transmembrane helices.

Beyond the Apicomplexa, LAP orthologues were identified in their closest living relatives: the chromerids (table 1; electronic supplementary material, table S1). The latter constitute a group of coral-associated photosynthetic algae whose plastids share many similarities with the apicoplast including four limiting membranes [16,17]. The Chromerida are considered to share a most recent common ancestor with the Apicomplexa, a view that is strongly supported by phylogenomics [18–21]. The two known species of chromerids, *Chromera velia* and *Vitrella brassicaformis*, together with their predatory relatives the colpodellids, form the chrompodellid lineage [16]. We identified putative LAP-encoding transcripts in the colpodellids (not shown), indicating that the LAPs are encoded across the Chrompodellida.

The analysis of the *V. brassicaformis* genome revealed that the chromerids possess the most extensive LAP repertoire encoding all LAP types (table 1; electronic supplementary material, table S1) plus an additional two LCCL-domain proteins not found in the Apicomplexa, each possessing a single LCCL domain downstream of five predicted transmembrane helices (figure 2*b*). Assuming that the latter interacts with other LAP members, as do LAPs 1–6 in *Plasmodium* [9,22], this protein could serve to anchor the LAP complex to cellular membranes in the chromerid lineage. Other membrane-bound proteins interacting with the LAP complex, such as NAD(P) transhydrogenase [9], may have made this protein obsolete in apicomplexan lineages like *Plasmodium*. In any event, the LAP repertoire found in chromerids indicates that the most recent common ancestor of the Apicomplexa and Chromerida already possessed genes for all the different LAP types, which subsequently spread to extant lineages by vertical transfer followed by lineage-specific gene duplication or loss.

Apicomplexa and chrompodellids—together with dinoflagellates, perkinsids and ciliates—form the Alveolata supergroup [23]. We did not find LAP orthologues in the latter three alveolate lineages. We could, however, identify intact transcripts encoding full-length LAP3 and LAP2/4 proteins in predatory colponemids (figure 2*a*; electronic supplementary material, table S1). The availability of only transcriptome shotgun assemblies (TSA) sequence data for this organism precludes a precise determination of its LAP repertoire. AlphaFold predictions of the three-dimensional structures of *Colponema vietnamica* LAP3 and LAP2/4 proteins revealed a very similar modular architecture to their apicomplexan counterparts (electronic supplementary material, figure S1), supporting structural conservation of the LAPs throughout evolution. The Colponemida were recently shown by phylogenomics to form a sister group to all other alveolates [24], and they thus represent an older lineage in which LAPs are present. We did not find LAP orthologues in other members of the TSAR (Telonemia, Stramenopila, Alveolata and Rhizaria) supergroup [25], or in any other eukaryotic lineages for that matter, indicating that the LAPs first evolved in the last common ancestor of Alveolata and Colponemida. Interestingly, the AlphaFold-predicted *C. vietnamica* LAP3 structure revealed two additional, unspecified domains of some 60 amino acids each on either side of the GAL domain (electronic supplementary material, figure S1), suggesting that the LAP3/5 predecessor was more complex than its current apicomplexan equivalents and providing further evidence for domain losses during its evolution.

### 2.3. LAP evolution

**Figure 3.**
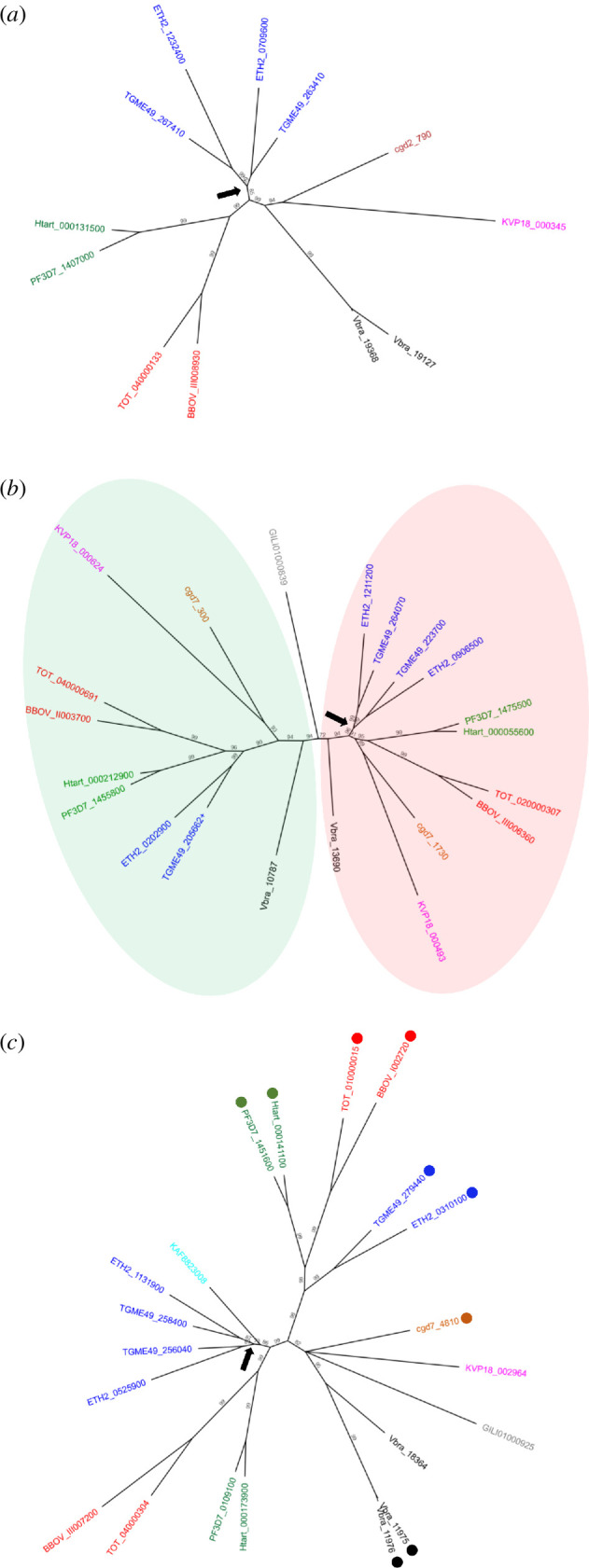
Evolution of LAP types. Bayesian inference-generated phylogenetic trees of LAP1 type (*a*), LAP2/4 type (*b*) and LAP3/5 type (*c*) amino acid sequences from chromerid (black), colponemid (grey), gregarine (pink), cryptosporidium (maroon), haemosporidian (green), piroplasmid (red), coccidian (blue) and nephromycid (cyan) lineages. LAP5 sequences in (*c*) are indicated with dots. Black arrows in (*a–c*) indicate gene duplication events in the coccidian lineage. In (*b*), differenty coloured backgrounds denote two distinct paralogous groups of LAP2/4 proteins. Numbers indicate branch support values.

To shine further light on the evolution of the LAPs, we carried out phylogenetic reconstructions of each of the LAP types separately. Very similar trees were obtained using Bayesian inference and maximum likelihood methods (figure 3; electronic supplementary material, figure S2). LAP1-, LAP2/4- and LAP3/5-type proteins clustered broadly according to established apicomplexan groupings (figure 3). The LAP1 tree revealed a chromerid-specific gene duplication event that has given rise to the two LAP1 paralogues in this lineage (figure 3*a*). Moreover, an independent Coccidia-specific *lap1* gene duplication (figure 3*a*; arrow) has given rise to the two LAP1 paralogues in the *Eimeriidae* and *Sarcosystidae* (table 1). In contrast to LAP1, the LAP2/4 phylogeny revealed two distinct radiations that have likely descended from ancestral LAP2/4 paralogues (figure 3*b*). A more recent gene duplication event specific to the coccidian lineage was revealed for one of these paralogues (figure 3*b*, black arrow), giving rise to the extra (third) LAP2/4 protein in the Coccidia compared with Hematozoa, Cryptosporidia or Gregarinia (table 1).

The LAP3/5 phylogeny showed LAP5 and LAP3 proteins separately clustering in Hematozoa and Coccidia (figure 3*c*), indicating they have descended from ancestral *lap5* and *lap3* genes, respectively, instead of having been derived from more recent gene duplications followed by loss/gain of a LCCL module. Indeed, this view is supported by the presence of both LAP3 and LAP5-encoding genes in chromerids (table 1). A chromerid-specific gene duplication appears to have given rise to the two LAP5 paralogues (Vbra_11975 and Vbra_11976) in this lineage (figure 3*c*). Their corresponding genes are tandemly located on the genome, pointing to a recent duplication event. A *lap3* gene duplication event can be identified in the Coccidia (figure 3*c*, black arrow) giving rise to the two LAP3 paralogues in this lineage. Based on available genomic sequence data, gregarine and cryptosporidian lineages have lost their *lap5* and *lap3* genes, respectively (figure 3*c*; table 1).

The two distinct ancestral origins of the LAP2/4 proteins are conserved across chromerids and apicomplexan lineages (figure 3*b*), suggesting that the two paralogues of this protein could have distinctive roles. Indeed, while *Plasmodium* LAP2 and LAP4 have the same loss-of-function phenotype [2,4], probably because they operate as part of the same LAP complex [7,9,22], it was shown in *P. berghei* that their specific interactions are different: LAP2 binds strongly to LAP3, whereas LAP4 interacts strongly with LAP5 [9,22]. This observation combined with the apparent descendants of LAP3 and LAP5 from ancestral *lap3* and *lap5* genes (figure 3*c*) indicates that these specific pairings could be ancestrally conserved.

Even though we did not find any LAP orthologues in the two largest alveolate lineages: dinoflagellates and ciliates, we identified a number of ‘LAP-like’ proteins in some dinoflagellates (*Symbiodiniaceae* and *Amoebophryaceae*), possessing different combinations of LAP modules including Ricin, F58C, SRCR and LCCL (electronic supplementary material, figure S3). It is tempting to speculate that these could be the products of rearrangements and modifications of the *lap* genes that were present in the ancestral dinoflagellate. Interestingly, a gene product identified in *Effrenium voratum* (CAJ1387072) contains, besides F58C and SRCR domains, two CPW-WPC domains (electronic supplementary material, figure S3). The CPW-WPC domain (IPR006387) is characterized by six conserved cysteine residues and six well-conserved aromatic sites specific to proteins from apicomplexans and chromerids [26]. In *Plasmodium,* CPW-WPC domain-containing proteins were recently shown to interact with the LAP complex with which they share a subcellular localization [9]. This is to our knowledge the first example of a protein that combines CPW-WPC domains with those found in LAPs, further supporting a functional link between these modules.

## 3. Discussion

Our studies presented here show that the LAPs are evolutionarily conserved from colponemids through to recent apicomplexans like *Plasmodium*. Colponemids have little in common with apicomplexans in terms of their biology: Apicomplexans are endosymbionts of animals with often parasitic relationships that possess an apical complex and gliding motility. In contrast, colponemids are free-living biflagellate eukaryovores that possess extrusive organelles (trichocysts and toxicysts) for active predation by phagocytosis, and their habitats appear restricted to freshwater environments [24]. The transition from phagocytosis to myzocytosis (tube feeding) is thought to have instigated the evolution of the apical complex (including conoid and secretory organelles) in the Myzozoa (i.e. alveolates excluding Ciliophora) [24,27]. The fact that colponemids do not feed by myzocytosis thus indicates that the LAPs pre-date the apical complex. In addition, plastid organelles appear to be ancestrally absent from colponemids [24,28], implying that the LAPs also pre-date the acquisition of the plastid through secondary endosymbiosis with a red alga [29]. Our results furthermore indicate that the LAPs, and possibly the molecular processes in which they are involved, have been lost in dinoflagellate, perkinsid and ciliate lineages.

The structural conservation of the LAPs despite their complex architectures points to conservation of their core functions. However, it is hard to identify a process that is shared between the biologically diverse colponemids, apicomplexans and chrompodellids, as well as being potentially lost in dinoflagellates, perkinsids and ciliates. In *Plasmodium*, where the LAPs have been best studied, the proteins have been located in the parasitophorous vacuole (PV) and host cell cytoplasm of *P. falciparum* gametocytes [4,30] and in the crystalloid—a multivesicular putative secretory organelle—in *P. berghei* ookinetes [10–12]. Moreover, genetic ablation of LAP expression abolished formation of crystalloids and blocked sporozoite development (*P. berghei*) or egress (*P. falciparum*) in the oocyst [2–4,11]. In *Cryptosporidium parvum*, LAP2 (Cpa135) was localized in apical secretory organelles of sporozoites as well as in the PV of sporozoite-infected host cells [31]. These combined observations point to a potential role of the LAPs in a vesicle-mediated exocytic pathway. Secretory organelles form part of both canonical and archetypal apical complexes found in Apicomplexa and related alveolate lineages, respectively [32], and the possible homology between them is of great interest. Determining the subcellular localization, for example, using specific antibodies, of *Plasmodium* LAP orthologues in deeper lineages such as colponemids could shed important new light on this.

## 4. Material and methods

LAP orthologue searches were conducted using *Plasmodium* LAPs as protein queries in BlastP searches of nonredundant protein databases, or TblastN searches of whole genome shotgun (WGS) and TSA databases via the National Centre for Biotechnology Information (NCBI) (https://ncbi.nlm.nih.gov) and the Eukaryotic Pathogen, Vector and Host Informatics Resource (VEuPathDB) (https://veupathdb.org) [33].

Domain searches were conducted on protein queries with the NCBI CD-Search tool [34] and Conserved Domain Database (CDD) [35], with InterProScan of the InterPro family of databases [36] (https://www.ebi.ac.uk/interpro) and with Simple Modular Architecture Research Tool (SMART) (https://smart.embl.de  ) [37]. Conserved protein modules were assigned when detected in at least one orthologue.

Predicted three-dimensional structures of *P. falciparum* LAPs were retrieved via the AlphaFold Protein Structure Database (https://alphafold.ebi.ac.uk) [38]. A comparison of query protein structures against a *P. falciparum* subset of the AlphaFold database was conducted using the AF-DB search option of the DALI server (http://ekhidna2.biocenter.helsinki.fi/dali) [39]. AlphaFold predicted three-dimensional structures of *C. vietnamica* LAP3 and LAP2/4 were generated in AlphaFold Colab (https://colab.research.google.com/github/deepmind/alphafold/blob/main/notebooks/AlphaFold.ipynb).

Phylogenetic analyses were conducted with Geneious Prime software. Phylogenies of combined LAP types were done with MUSCLE multiple alignment and FastTree tree building. Phylogenetic reconstruction of LAPs by type was carried out with Clustal Omega multiple alignment [40] followed by Bayesian inference (MrBayes 3.3.2) [41] or maximum likelihood (PhyML 3.3) [42] methods using Jones–Taylor–Thornton substitution models.

## Data Availability

Supplementary material is available online [43].
